# Clinical Utility of Broad-Range PCR Testing and Impact on Outcomes in Adults with Suspected Infection †

**DOI:** 10.3390/antibiotics13121166

**Published:** 2024-12-03

**Authors:** Kaitlyn Weinert-Stein, Mackenzie Cater, Sree Sarah Cherian, Reem Azem, Ana E. Khazan, Ankita P. Desai, LeAnne Tripp, Peter Paul Lim, Lisa M. Stempak, Leila S. Hojat

**Affiliations:** 1 Department of Medicine, Case Western Reserve University School of Medicine, Cleveland, OH 44106, USAmackenzie.cater@uhhospitals.org (M.C.);; 2 Department of Medicine, Cleveland Clinic, Cleveland, OH 44195, USA; 3 Department of Pediatrics, Case Western Reserve University School of Medicine, Cleveland, OH 44106, USA; 4 Department of Clinical Pathology, Case Western Reserve University School of Medicine, Cleveland, OH 44106, USA; 5 Department of Clinical Pathology, University Hospitals Cleveland Medical Center, Cleveland, OH 44106, USA; 6 Department of Pediatrics, University Hospitals Rainbow Babies and Children’s Hospital, Cleveland, OH 44106, USA; 7 Department of Pediatric Pharmacy, University Hospitals Rainbow Babies and Children’s Hospital, Cleveland, OH 44106, USA; 8 Department of Pediatrics, Avera McKennan University Health Center, Sioux Falls, SD 57106, USA; 9 Department of Pediatrics, University of South Dakota Sanford School of Medicine, Sioux Falls, SD 57069, USA; 10 Department of Clinical Pathology, Cleveland Clinic, Cleveland, OH 44195, USA; 11 Department of Medicine, University Hospitals Cleveland Medical Center, Cleveland, OH 44106, USA

**Keywords:** broad-range PCR, molecular diagnostics, diagnostic stewardship, antimicrobial stewardship

## Abstract

**Background**: The development of broad-range polymerase chain reaction (BR-PCR) and next-generation sequencing techniques has significant implications for antimicrobial stewardship by increasing clinicians’ ability to provide a tailored antimicrobial regimen. We sought to explore the clinical utility of BR-PCR testing and its impact on antimicrobial treatment among an adult cohort in a large hospital system. **Methods**: We retrospectively evaluated samples that underwent BR-PCR testing between 2017 and 2021 and summarized their clinical characteristics and impact on antimicrobial therapy. We identified BR-PCR testing as having clinical utility if the results led to adjustment of antimicrobial choice or duration or to confirmation of the initial empiric regimen, while no clinical utility was assigned to results that were negative or clinically insignificant, unavailable due to loss to follow-up, or lacking clinical indication (treatment completed before the test results returned or conventional cultures revealed the causative pathogen). **Results**: Among 359 specimens, BR-PCR was positive for 107 (30%). Clinical utility was identified for 106 (29.5%) specimens, including 45 with negative BR-PCR results. The rates of clinical utility varied based on the type of sample tested, with the highest clinical utility associated with cranial samples (60%), followed by body fluid (56%) and endovascular (54%) samples, and the lowest with CSF (15%) and bone and joint (19%) samples. We also identified many BR-PCR tests that were not clinically indicated (23.4%). **Conclusions**: This study highlights the utility of BR-PCR testing to support antimicrobial stewardship initiatives. Further studies are needed to identify clinical scenarios in which it is appropriate to order BR-PCR testing and for a careful interpretation of negative BR-PCR results.

## 1. Introduction

Reliable microbiologic data are essential in the field of infectious diseases not only for an accurate diagnosis of infectious syndromes, but also for the optimization of the antimicrobial therapy. Unfortunately, when microbiologic data are not available, providers must often resort to expensive, broad-spectrum antimicrobial treatment regimens that contribute to the development of antimicrobial resistance and can lead to adverse events such as *Clostridioides difficile* infection, nephrotoxicity, hypersensitivity reactions, hematologic abnormalities, and hepatic dysfunction [1,2]. Faced with the looming threat of increasing antimicrobial resistance further exacerbated by the COVID-19 pandemic, providers must appropriately utilize the available antimicrobial stewardship tools to identify the most effective antimicrobial regimen with the fewest short- and long-term consequences [3,4].

Culture-based methods for pathogen identification and antimicrobial susceptibility testing have traditionally been considered the gold standard for guiding appropriate antimicrobial selection; however, culture-based methods have their limitations. For example, cultures are reliant on the quality of the available samples, and yield can decrease with insufficient specimen volume, poor specimen collection, handling or transport, low organism concentration, and pre-treatment with antimicrobials [5]. Additionally, certain organisms are more fastidious and do not readily grow in culture [6]. Scientific advances have led to the availability of newer methods for detecting infectious organisms, such as broad-range polymerase chain reaction (BR-PCR) and next-generation sequencing [7]. These methods aid in identifying organisms when culture and serologic methods cannot and thus provide the necessary guidance on the management of various conditions.

BR-PCR testing consists of gene amplification followed by sequencing. A common form of BR-PCR for bacterial identification uses the 16S ribosomal RNA gene, one of the primary targets in bacterial taxonomic identification. Given that this region is highly conserved and hypervariable, reliable primers can be developed that allow for a consistent detection of different bacteria via primer differentiation [8]. This modality is used both clinically and in phylogenetic studies [9]. Similar BR-PCR techniques are used for the identification of fungal pathogens, involving the 28S rRNA gene and Internal Transcribed Spacer (ITS) regions. Multiple gene targets may be used for the identification of mycobacteria, including heat shock protein (hsp65), RNA polymerase (rpoB), and 16S rRNA genes. The advantages of BR-PCR sequencing include the detection of a wide spectrum of organisms, the ability to detect both viable and nonviable pathogens, and species differentiation [10]. Some limitations include the costly equipment, longer turnaround times relative to other methods, and the lack of antimicrobial susceptibility data [11].

Despite its increasing utilization and potential stewardship applications, limited studies have investigated the impact of BR-PCR sequencing on diagnostic accuracy and clinical decision-making [12,13]. Prior studies evaluated the sensitivity and specificity as well as positive and negative predictive values of BR-PCR for informing diagnostic utility [14,15]. Additionally, studies assessed how a positive test result may inform clinical decisions such as antimicrobial de-escalation [16,17,18]. Building upon our prior analysis among a pediatric population and expanding on our original analysis of the adult data, we sought to provide insight on the clinical impact of BR-PCR testing in a larger sample of adult patients [19,20]. Specifically, we focused on describing whether BR-PCR testing was associated with de-escalation or discontinuation of antimicrobial therapy in patients with bacterial, fungal, and mycobacterial infections. Additional explored metrics included longer-term outcomes such as adverse effects of antimicrobials and infection-related readmissions rates.

## 2. Results

A total of 366 clinical specimens from 352 unique patients were reviewed; after the exclusion of specimens from the same site, 359 specimens were included in the analysis. The BR-PCR results identified an organism in 107 (29.8%) of the specimens (Table 1). Bacterial BR-PCR was the most frequently performed and correlated with the highest rate of positivity (28.3%), followed by fungal PCR (13.5%) and mycobacterial PCR (7.5%). Concurrent conventional cultures of the same specimens were positive in 79 (22%) of the cases; of these, 24 (30.4%) provided results in concordance with the PCR results.

**Figure 1:**
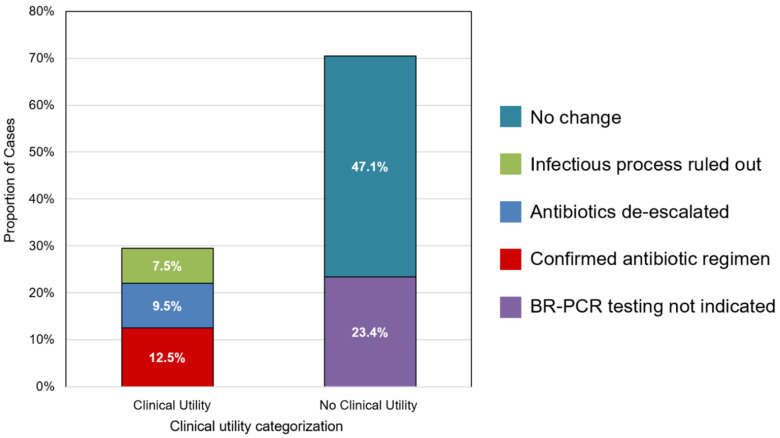
Proportion of clinical specimens stratified by clinical impact of BR-PCR sequencing. Percentage represents proportion of subcategory of clinical utility relative to entire cohort (*n* = 359).

Clinical utility was identified in 106 (29.5%) of all specimens, including 45 specimens with negative BR-PCR results. De-escalation of the antimicrobial therapy and confirmation of the antimicrobial regimen occurred for 9.5% (*n* = 34) and 12.8% (*n* = 46) of the specimens, respectively (Figure 1). Additionally, 7.5% (*n* = 27) of the specimens were designated as having clinical utility related to discontinuation of antimicrobials, which primarily occurred in cases with a negative BR-PCR; in only two cases, antimicrobials were discontinued due to the provider’s suspicion of the identified organism representing a contaminant.

**Figure 2:**
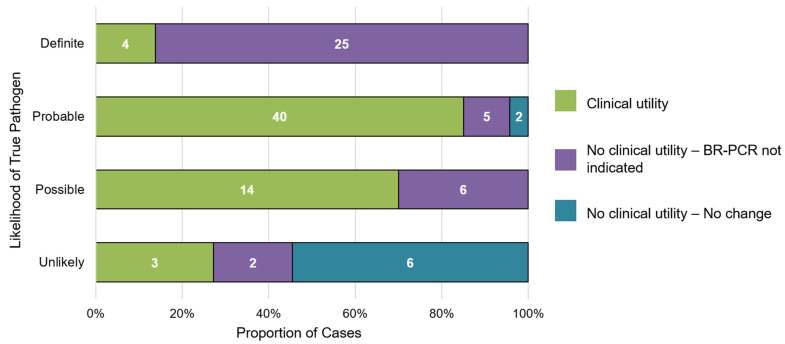
Likelihood of a positive BR-PCR result (*n* = 107) representing a true pathogen and associated clinical utility. Data labels within bars represent number of clinical specimens classified into each clinical utility category, stratified by pathogenicity category (i.e., definitive, probable, possible, unlikely, as defined in Methods Section 4.4).

A total of 84 specimens (23.4%) were not clinically indicated, primarily due to the BR-PCR results returning after a positive conventional culture result. Among these 84 specimens, 48 had positive culture results, which, for 23 of them, were concordant with the BR-PCR results. An additional six cases had positive culture results for alternative clinical specimens. Of the remaining 169 specimens without clinical impact, nearly all had negative BR-PCR results that did not influence management (*n* = 161). The remainder had discordant cultures or clinically insignificant results or were lost to follow-up. Among the patients with a positive BR-PCR result including those with negative conventional culture results (65 specimens), stratified by likelihood of the identified organism(s) representing a true pathogen, clinical utility was most likely for organisms representing probable (85.1%) or possible (70%) true pathogens (Figure 2). Only 13.8% of the definite pathogens and 27.3% of the unlikely pathogens had results which provided clinical utility.

**Figure 3:**
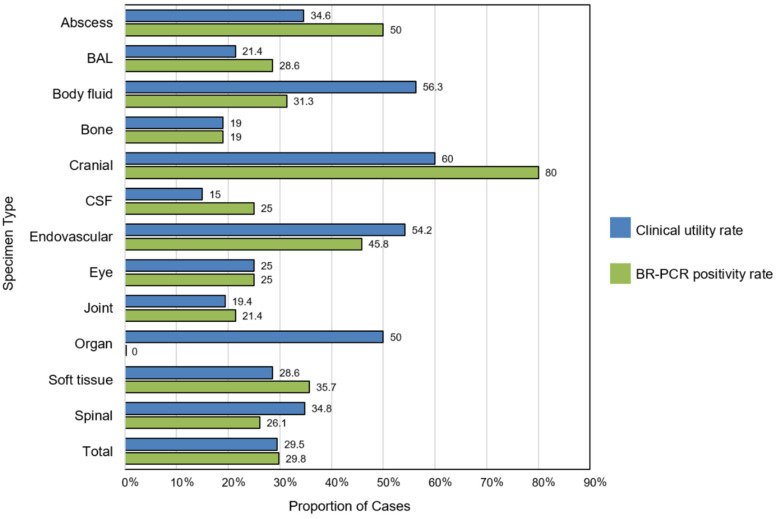
Clinical utility of BR-PCR results and BR-PCR positivity rate by clinical specimen type. Abbreviations: BAL, bronchoalveolar lavage; CSF, cerebral spinal fluid.

The most frequently tested specimen types were joint (27.3%, *n* = 98), abscess (14.5%, *n* = 52), spinal tissue (12.8%, *n* = 46), and bone (11.7%, *n* = 42) (Table 1). Assessing the performance of BR-PCR sequencing, cranial specimens other than CSF included the highest proportion of results with clinical utility (60%, *n* = 3), followed by body fluid specimens (56.3%, *n* = 9), and endovascular specimens (54.2%, *n* = 13) (Figure 3). The specimen types with lowest clinical utility included CSF (15%, *n* = 3), bone (19%, *n* = 8), and joint (19.4%, *n* = 19).

Among the clinically significant results, the most common categories of organisms detected were typical skin commensals (19.8%, *n* = 21 specimens), followed by Gram-positive organisms (14.2%, *n* = 15 specimens), and anaerobes (7.5%, *n* = 8 specimens) (Table 2). The most frequently identified individual species were *Staphylococcus epidermidis* (8), *Cutibacterium acnes* (7), and *Fusobacterium* spp. (6). Six specimens identified more than one organism, predominantly including the oral flora, such as *Fusobacterium* spp. and *Prevotella* spp., and the skin flora.

We analyzed outcomes among the patients after excluding those lost to follow-up, identifying a total of 330 patients including 102 with BR-PCR results of clinical utility and 228 with BR-PCR results of no clinical utility. Adverse events, infection-related readmission within 90 days, and infection-related mortality within 90 days were all numerically lower but not statistically significant in the clinical utility group (Table 3). Adverse events attributed to antimicrobials experienced by the patients included infusion reaction, drug reaction with eosinophilia and systemic symptoms, blood dyscrasias, encephalopathy, seizures, renal insufficiency, gastrointestinal intolerance, *C. difficile* infection, mucocutaneous and vulvovaginal candidiasis, myopathy, and line-associated complications.

## 3. Discussion

The existing literature regarding the clinical utility of BR-PCR testing largely focuses on the utility of positive BR-PCR results to identify a pathogen and guide antimicrobial management decisions, as occurred in 61 cases (17%) in our study with positive results observed to have clinical utility. However, our study additionally identified 45 (12.5%) cases with negative BR-PCR results that were determined to be of clinical utility. These findings are comparable to the findings of Lim et al. in a recent analysis of BR-PCR testing in a pediatric population, who found that 24.6% of negative BR-PCR results were clinically useful [19]. Building on this background, our results speak to the potential clinical utility of negative as well as of positive BR-PCR results.

While positive BR-PCR results were more likely to be clinically useful than negative results (57% of the positive results had clinical utility compared to 17.9% of the negative results), negative results that are clinically useful are particularly important from an antimicrobial stewardship perspective. In this study, clinically useful negative results were used to inform decisions regarding antimicrobial discontinuation, antimicrobial de-escalation, and shortening of antimicrobial duration, bolstering antimicrobial stewardship efforts. As BR-PCR testing has become more well-known and more widely utilized, it may be that clinicians have increasing confidence in using negative BR-PCR results to rule out particularly virulent pathogens (leading to antimicrobial de-escalation) or infectious pathologies as a whole (leading to antimicrobial discontinuation). In the majority of the cases, the patients had already received an empiric antimicrobial regimen for several days or weeks by the time that their BR-PCR results returned from the reference laboratory, which may also have caused clinicians to feel more comfortable using a negative BR-PCR result to shorten the duration of or discontinue the antimicrobial therapy. It is important to note that decisions regarding the discontinuation of antimicrobial therapy should be based on the clinician’s determination of the likelihood of infection as well as of the clinical context of the patient as a whole, not determined by the BR-PCR result alone.

Our study reinforces the findings of previous studies in that the degree of BR-PCR clinical utility varied based on the type of sample sent for testing. Endovascular specimens had clinical utility in 54.2% of the cases, in contrast to joint specimens that showed clinical utility in 19.4% of the cases, as determined through the 16S rRNA results. This correlates with the patterns observed by both Fida et al. (22% of the results from cardiovascular samples leading to changes in management compared to 6% of the results from musculoskeletal samples) and Akram et al. (37.5% of cardiovascular results leading to changes in management compared to 15% for joint samples) [14,17]. Both of these studies found higher likelihoods of a positive BR-PCR result from the cardiovascular tissue than from other sample types, which could lead to the higher rate of clinical utility observed with these sample types. Our study also found that the BR-PCR results from CSF were the least likely to be clinically useful among the results from all sample types (only 15% of the results were clinically useful), similar to the findings of Fida et al. (only 5% of the results from CSF led to changes in management) and Kerkhoff et al. (only 1.5% of the results) [16]. Of note, we included positive BR-PCR results that confirmed an already selected empiric antimicrobial regimen as clinically useful in addition to BR-PCR results that led to a change in management, whereas other studies only considered those BR-PCR results that caused a change in management, which contributed to the higher rates of clinical utility observed in our study compared to others. This study also found a significant percentage of not clinically indicated BR-PCR tests (23.4%), deemed not indicated due to the fact that either a positive conventional culture returned prior to the BR-PCR result or the set empiric course of antimicrobial therapy was already completed by the return of the BR-PCR result. This finding highlights the importance of continuing education related to BR-PCR testing to enable clinicians to weigh the expected turnaround time of the test itself against the duration of the proposed empiric antimicrobial regimen. It also underscores the importance of institutional expert guidance and/or criteria surrounding the ordering of BR-PCR testing, such as requiring the approval of a microbiology laboratory director or infectious disease specialist prior to ordering. Even in institutions with a short time-to-result if BR-PCR is available on site, testing may not be indicated and should be performed based on guidance from local experts. At our institution, the infectious disease consult service is involved in nearly all cases in which BR-PCR testing is performed, and testing was carried out at the discretion of the attending consultant, with all cases reviewed for appropriateness by a microbiology laboratory director. Further study is needed to clarify the ideal length of time to wait for conventional culture growth prior to sending samples for BR-PCR testing, as well as regarding the best methods of storage to preserve the sample material during this time period. Identifying an optimal waiting period would aid in reducing the number of BR-PCR results with no clinical indication due to a prior positive conventional culture, thereby leading to cost savings and a decrease in the duplication of effort in the laboratory [21].

Regarding the outcomes, we identified numerically lower rates of adverse events, readmission, and mortality in the clinical utility group; however, none of these results were statistically meaningful. Notably, our study included 56 patients who were lost to follow-up after BR-PCR was sent. Since the patients lost to follow-up could not be contacted regarding their BR-PCR result, no changes could be made to their antimicrobial regimen even if warranted, thereby negating the utility of the BR-PCR testing. Significant measures should be taken to prevent patients from being lost to follow-up, not only those whose BR-PCR test has been sent and is pending at the time of discharge, but all patients.

The limitations of this study, a retrospective chart review, include the possibility that incomplete or unclear clinical documentation could have led to misclassification of the clinical utility of individual BR-PCR results. We did not record assessments of inflammation (serologic such as leukocytosis, ESR, and CRP or pathologic findings), which could have influenced clinicians’ decision-making related to antimicrobial de-escalation or discontinuation and the shortening of antimicrobial regimens. Additionally, BR-PCR testing is a send-out test at our institution, with a turn-around time of approximately one week. This extended turn-around time may have led to an over-estimation of the clinical significance of negative BR-PCR results, as clinicians may have felt more comfortable discontinuing an antimicrobial regimen with a longer portion of the antimicrobial course already completed. Finally, due to the number of patients lost to follow-up, our study may have been under-powered to detect a difference in clinical outcome endpoints such as infection-related readmission rate and mortality.

## 4. Methods

We performed a retrospective cohort study of adults admitted to a hospital system in northeast Ohio, USA comprising 15 medical centers. The Institutional Review Board of University Hospitals of Cleveland approved our study as an expedited protocol (IRB STUDY20211398).

### 4.1. Patient Population and Specimens

We included hospitalized adult patients (≥18 years old) for whom BR-PCR sequencing was performed on clinical specimens between June 2017 and October 2021. The patients were identified by a manual review of requisition forms retained for specimens sent to the University of Washington (Seattle, WA, USA) for BR-PCR sequencing. The specimens were organized by site (Table 4). Specimens from different sites for the same patient were counted as individual specimens in the analysis, and only the first collected sample was counted from the same site.

### 4.2. Broad-Range PCR Processing Methods

The samples underwent BR-PCR sequencing at the University of Washington Molecular Diagnostics Laboratory. The clinical samples were analyzed by direct detection of microbial DNA from tissues; DNA was isolated and extracted from the patient specimens and amplified by conventional PCR using broad-range primers: the 16S rRNA gene for bacterial pathogens, the 28S rRNA and ITS genes for fungal pathogens, and the hsp65, rpoB, and 16S rRNA genes for mycobacterial pathogens. Amplification was followed by sequencing and a comparison of the sequences against institutional and publicly available DNA sequences for an accurate identification of organisms [22]. A detailed description of BR-PCR sequencing was previously published in the literature [23]. Polymicrobial specimens unable to be resolved by the previous methods underwent next-generation DNA sequencing.

### 4.3. Clinical Data Review and Outcomes

Clinical information including demographics, primary infection diagnosis, specimen type, conventional cultures, and antimicrobial therapy was obtained via a chart review of electronic medical records. Documentation by infectious disease specialists was reviewed to determine the definitive choice and duration of the antimicrobial therapy and if the BR-PCR results affected the decision. Adverse events attributed to the antimicrobial therapy, readmission to the hospital within 90 days for the same infection or a related complication, and infection-related mortality at 90 days were evaluated as outcomes.

### 4.4. Analysis

We performed a descriptive analysis of specimen types and organisms identified by BR-PCR. The BR-PCR results that identified an organism were further adjudicated to determine the likelihood of representing a true pathogen. This was evaluated by concordance with conventional culture results, inherent microorganism pathogenicity, and clinical scenarios including infectious diseases assessment. The categories of likelihood of pathogenicity included the following: definite, referring to organisms which were also identified in conventional culture (either the same or a separate specimen compared to that identified by BR-PCR); probable, including organisms that would be considered typical pathogens for the suspected clinical syndrome; possible, referring to cases in which the identified organism could have represented a contaminant but was of enough clinical concern that treatment was recommended; and unlikely, including organisms generally consistent with contamination, organisms typically found as commensals, or polymicrobial specimens for which treatment was not recommended.

The specimens were additionally stratified into clinical utility and no clinical utility categories based on the provider’s interpretation of the results (Table 5). Clinical utility was assigned to results that influenced the provider’s decision-making, with adjustment in the antimicrobial choice, adjustment in antimicrobial duration, or confirmation of the initial empiric antimicrobial treatment regimen. The no-clinical-utility category was assigned to negative or clinically insignificant results (i.e., contaminants or artifacts), results that could not be acted upon due to the patient being lost to follow-up, or testing that was not indicated per institutional guidelines. Due to the prolonged time-to-result of the BR-PCR testing performed as a send-out test in our health system, recommendations are to send testing only if treatment is expected to continue for longer than 14 days and if conventional culture results are negative. The clinical utility results were stratified by PCR type, positivity, and specimen type and analyzed for impact on the pre-specified outcomes of interest. The outcomes were analyzed using Fisher’s exact test, excluding patients lost to follow-up. Differences were considered statistically significant at *p* < 0.05. Statistical analyses were performed in R version 4.2.3.

## 5. Conclusions

This study highlights the clinical utility of BR-PCR testing as a diagnostic tool to aid providers in selecting targeted antimicrobial regimens that promote antimicrobial stewardship and reduce patients’ risks of developing resistant pathogens or adverse effects from empiric antimicrobial regimens. More work must be done to support clinicians in identifying scenarios that are not appropriate for BR-PCR testing, such as in the case of empiric treatment durations that are shorter than the expected turnaround time for a BR-PCR result. Clinicians should also be aware of significant variability in the likelihood of clinical utility associated with the type of tissue being sampled. Our study indicates that negative BR-PCR testing can inform decision-making regarding antimicrobial discontinuation, though these are complex decisions, and more studies are needed to clarify the specific role of BR-PCR in guiding these decisions.

## Figures and Tables

**Table 1:** Summary of specimen types, results, and clinical utility.

Characteristic	Total Specimens	PCR-Positive Specimens	PCR Positivity Rate	Culture-Positive Specimens	Cultures in Concordance with PCR ^a^	Specimens with Clinical Utility	Clinical Utility Rate
	*n* (%)	*n*	%	*n* (%)	*n* (%)	*n*	%
All specimens	359 (100)	107	29.8	67 (18.7)	25 (37.3)	106	29.5
PCR type ^b^							
Bacterial	346 (96.4)	98	28.3	33 (9.5)	22 (66.7)	98	28.3
Fungal	52 (14.5)	7	13.5	3 (5.8)	2 (66.7)	20	38.5
AFB	40 (11.1)	3	7.5	2 (5)	2 (100)	14	35
PCR result							
Positive	107 (29.8)	107	100	36 (33.6)	25 (69.4)	61	57
Negative	252 (70.2)	0	0	31 (12.3)	NA	45	17.9
Specimen type							
Abscess	52 (14.5)	26	50	14 (26.9)	7 (50)	18	34.6
BAL	14 (3.9)	4	28.6	4 (28.6)	0 (0)	3	21.4
Body fluid	16 (4.5)	5	31.3	1 (6.3)	0 (0)	9	56.3
Bone	42 (11.7)	8	19	8 (19)	2 (25)	8	19
Cranial	5 (1.4)	4	80	1 (20)	1 (100)	3	60
CSF	20 (5.6)	5	25	3 (15)	2 (66.7)	3	15
Endovascular	24 (6.7)	11	45.8	7 (29.2)	0 (0)	13	54.2
Eye	4 (1.1)	1	25	0 (0)	NA	1	25
Joint	98 (27.3)	21	21.4	22 (22.4)	9 (40.9)	19	19.4
Organ	10 (2.8)	0	0	2 (20)	NA	5	50
Soft tissue	28 (7.8)	10	35.7	9 (32.1)	1 (11.1)	8	28.6
Spinal	46 (12.8)	12	26.1	7 (15.2)	3 (42.9)	16	34.8

Abbreviations: AFB, acid-fast bacilli; BAL, bronchoalveolar lavage; CSF, cerebral spinal fluid. ^a^ Applies to specimens with positive culture results only. Specimen types with no PCR-positive or no culture-positive results labeled as “NA”. ^b^ Total PCR type number greater than specimen number due to multiple PCR types sent for certain specimens.

**Table 2:** Organisms identified through BR-PCR results by category stratified by clinical utility. Individual species and subtypes with count in parentheses shown for specimens with clinical utility only (*n* = 106).

Identified Organism Categories and Species/Subtypes	Clinical Utility (*n* = 106)		No Clinical Utility (*n* = 253)	
	*n*	%	*n*	%
Negative	45	42.5	207	81.8
Polymicrobial	6	5.7	9	3.6
Gram-positive cocci	15	14.2	12	4.7
Beta-hemolytic streptococci (7), *S. pneumoniae* (2), Viridans group streptococci (3), *Granulicatella adiacens* (1), *Staphylococcus aureus* (1), *Enterococcus faecalis* (1)				
Gram-negatives	6	5.7	6	2.4
Enterobacterales (2), *Haemophilus influenzae* (2), *Capnocytophaga canimorsus* (1), *Bartonella* spp. (1)				
Skin Flora	21	19.8	13	5.1
*Staphylococcus epidermidis* (8), other coagulase-negative staphylococci (1), *Corynebacterium striatum* (1), other *Corynebacterium* spp. (2), *Cutibacterium acnes* (7), *Finegoldia magna* (2)				
Anaerobes	8	7.5	2	0.8
*Fusobacterium nucleatum* (4), *Fusobacterium necrophorum* (2), *Porphyromonas endodontalis* (1), *Prevotella buccae* (1)				
Fungi	4	3.8	2	0.8
*Aspergillus fumigatus* (1), *Blastomyces dermatiditis* (1), *Candida albicans* (1), *Scedosporium boydii* (1)				
Acid-fast bacilli	1	0.9	2	0.8
*Mycobacterium fortuitum* (1)				

**Table 3:** Comparison of outcomes between clinical utility and no clinical utility groups, excluding 56 patients who were lost to follow-up (*n* = 330). Abbreviation: CI, confidence interval.

Outcome	Clinical Utility *n* = 102	%	No Clinical Utility *n* = 228	%	*p*-Value (95% CI)
Adverse events	17	16.7	49	21.5	0.37 (0.37–1.38)
Readmission	10	9.8	41	18	0.07 (0.21–1.07)
Mortality	2	2	11	4.8	0.36 (0.04–1.86)

**Table 4:** Sample type categories and subcategories.

Sample Type Category	Sample Type Subcategory
Body Fluid	Pericardial fluid; Peritoneal fluid; Pleural fluid/Pleura; Middle ear fluid; Cyst/Seroma fluid
Cranial	Dural tissue; Subgaleal tissue; Epidural tissue
Endovascular	Valve; Endovascular Graft
Organ	Lung; Kidney; Liver; Brain; Lymph node
Abscess	Abscess; Pus
Spinal Tissue	Vertebral bone; Disc tissue; Intervertebral space
Soft Tissue	Skin; Soft tissue; Fascia; Muscle biopsy
Eye	Vitreous fluid; Conjunctiva
Bronchoalveolar Lavage (BAL)	BAL; Tracheal aspirate
Joint	Joint tissue; Synovial fluid; Prosthetic joint
Bone	Non-vertebral bone
Cerebrospinal Fluid (CSF)	CSF

**Table 5:** Categories of clinical impact and associated inclusion criteria.

Clinical Impact Categories		Inclusion Criteria
Clinical utility	De-escalation of antimicrobials	Antimicrobial coverage was narrowed
		Duration of antimicrobial therapy was shortened
	Confirmation of empiric antimicrobial regimen	Antimicrobial coverage stayed the same or was escalated
		Duration of antimicrobial therapy stayed the same or was lengthened
	Infectious process ruled out	Antimicrobial therapy discontinued given low suspicion for infection
No clinical utility	No change in management (true negative utility)	Negative or clinically insignificant BR-PCR result AND antimicrobial regimen remained the same
		Patient lost to follow-up
	BR-PCR testing not indicated	Positive culture result before BR-PCR result
		Intended therapy less than or equal to 14 days

## Data Availability

Data from this study are not publicly available but may be available from the corresponding author upon reasonable request.
